# Modified Glasgow Prognostic Score is Associated With Risk of Recurrence in Bladder Cancer Patients After Radical Cystectomy: A Multicenter Experience

**DOI:** 10.1097/MD.0000000000001861

**Published:** 2015-10-23

**Authors:** Matteo Ferro, Ottavio De Cobelli, Carlo Buonerba, Giuseppe Di Lorenzo, Marco Capece, Dario Bruzzese, Riccardo Autorino, Danilo Bottero, Antonio Cioffi, Deliu Victor Matei, Michele Caraglia, Marco Borghesi, Ettore De Berardinis, Gian Maria Busetto, Riccardo Giovannone, Giuseppe Lucarelli, Pasquale Ditonno, Sisto Perdonà, Pierluigi Bove, Luigi Castaldo, Rodolfo Hurle, Gennaro Musi, Antonio Brescia, Michele Olivieri, Amelia Cimmino, Vincenzo Altieri, Rocco Damiano, Francesco Cantiello, Vincenzo Serretta, Sabino De Placido, Vincenzo Mirone, Guru Sonpavde, Daniela Terracciano

**Affiliations:** From the Division of Urology, European Institute of Oncology, Milan, Italy (MF, OD, DB, AC, DVM, GM, AB); Department of Urology, “Iuliu Hatieganu” University of Medicine and Pharmacy, Cluj-Napoca, Romania (OD); Genitourinary Cancer Section, Medical Oncology Division, University Federico II, Napoli, Italy (CB, GD, SD); Division of Urology, University “Federico II”, Naples, Italy (MC, VM); Department of Public Health, University “Federico II”, Naples, Italy (DB); Urology Institute, University Hospitals, Cleveland, OH, USA (RA); Department of Biochemistry, Biophysics and General Pathology, Second University of Naples, Naples, Italy (MC); Department of Urology, University of Bologna, S. Orsola-Malpighi Hospital, Bologna, Italy (MB); Division of Urology, University “La Sapienza”, Rome, Italy (ED, GMB, RG); Department of Emergency and Organ Transplantation, Urology, Andrology and Kidney Transplantation Unit, University of Bari, Bari, Italy (GL, PD); Division of Urology, IRCCS Fondazione G. Pascale, Napoli, Italy (SP); Department of Urology, Tor Vergata University of Rome, Rome, Italy (PB, LC, RH); Institute of Genetics and Biophysics, National Research Council (CNR), Naples, Italy (MO, AC); Department of Urology, University of Salerno, Salerno, Italy (VA); Department of Urology, Magna Graecia University, Catanzaro, Italy (RD, FC); Department of Surgical, Oncological and Stomatological Sciences, Institute of Urology, University of Palermo, Palermo, Italy (VS); Urologic Oncology, Division of Hematology and Oncology, Department of Medicine, University of Alabama, Birmingham, AL, USA (GS); Department of Translational Medical Sciences, University “Federico II”, Naples, Italy (DT).

## Abstract

Recently, many studies explored the role of inflammation parameters in the prognosis of urinary cancers, but the results were not consistent. The modified Glasgow Prognostic Score (mGPS), a systemic inflammation marker, is a prognostic marker in various types of cancers. The aim of the present study was to investigate the usefulness of the preoperative mGPS as predictor of recurrence-free (RFS), overall (OS), and cancer-specific (CSS) survivals in a large cohort of urothelial bladder cancer (UBC) patients.

A total of 1037 patients with UBC were included in this study with a median follow-up of 22 months (range 3–60 months). An mGPS = 0 was observed in 646 patients (62.3%), mGPS = 1 in 297 patients (28.6 %), and mGPS = 2 in 94 patients (9.1%).

In our study cohort, subjects with an mGPS equal to 2 had a significantly shorter median RFS compared with subjects with mGPS equal to 1 (16 vs 19 months, hazard ratio [HR] 1.54, 95% CI 1.31–1.81, *P* < 0.001) or with subjects with mGPS equal to 0 (16 vs 29 months, HR 2.38, 95% CI 1.86–3.05, *P* < 0.001). The association between mGPS and RFS was confirmed by weighted multivariate Cox model. Although in univariate analysis higher mGPS was associated with lower OS and CSS, this association disappeared in multivariate analysis where only the presence of lymph node-positive bladder cancer and T4 stage were predictors of worse prognosis for OS and CSS.

In conclusion, the mGPS is an easily measured and inexpensive prognostic marker that was significantly associated with RFS in UBC patients.

## INTRODUCTION

Worldwide, urothelial bladder cancer (UBC) is the 7th most frequent cancer in men and the 17th most frequent cancer in women, with annual mortality rates varying approximately in the range of 1 to 5 deaths per 100,000 men and 0.5 to 1.5 deaths per 100,000 women.^1^ Bladder cancer has been associated with a number of factors, including smoking, occupational exposure, pollution, as well as urinary calculi.^1,2^ Accurate prognostic assessment after radical surgery, which is the mainstay of treatment of localized disease, is important to define indication for adjuvant therapy.^3^ Multiple prognostic models with satisfactory accuracy have been proposed to evaluate the risk of recurrence or death in bladder cancer patients, but not all the variables included are readily measurable and most models have not been externally validated.^4^ Among the multitude of clinical, pathological, radiological, and genetic variables of potential clinical utility, serum markers of systemic inflammation may be particularly useful, because their intrinsic prognostic value is coupled to the inexpensiveness, simplicity, and lack of invasiveness of their assessment.^4^ Systemic inflammation, which has a detrimental effect in most solid tumors, can be measured by assessing circulating myeloid-derived suppressor cells, neutrophil count, white cell count, neutrophil–lymphocyte ratio (NLR), and C-reactive protein (CRP).^5–7^ The NLR measured at the time of cystectomy is associated with overall and cancer-specific mortality.^7^ CRP has an established prognostic role in UBC^8^ and has been extensively investigated in a variety of clinical settings, that is in patients undergoing transurethral resection of the tumor,^9^ chemo-radiotherapy,^10^ or radical cystectomy (RC)^11–13^ for loco-regional disease and in those with advanced recurrent/systemic disease.^14–16^ This acute phase response protein, which is released by hepatocytes in response to inflammatory cytokines and also by tumor cells themselves, can facilitate cancer cell survival by pleiotropic effects and can be accurately measured in the serum.^8^ CRP has been combined with albumin, which has an independent prognostic value in patients undergoing RC for UBC,^17^ in order to calculate the modified Glasgow Prognostic Score (mGPS), a well-characterized scale with independent prognostic value in various malignancies.^18^ As the clinical utility of the mGPS in the particular case of UBC patients is yet to be extensively explored,^19^ we aimed to investigate its prognostic value at the time of RC in a large retrospective cohort of patients with histologically confirmed UBC.

## PATIENTS AND METHODS

### Inclusion Criteria

Medical records of patients undergoing RC plus pelvic lymphadenectomy between January 2008 and December 2013 at 8 Italian urologic centers (University of Naples “Federico II”; “Pascale” National Cancer Institute of Naples; European Oncologic Institute, Milan; University of Bari “Aldo Moro”; University of Rome “La Sapienza”; University of Salerno; University of Catanzaro, Magna Graecia; and Humanitas Clinical and Research Center, Milan) were reviewed. Patients were included in this retrospective analysis if: they had histologic diagnosis of UBC; they had not received any neoadjuvant radiotherapy or chemotherapy; they did not present any infection or any other serious systemic inflammatory condition such as ischemia, acute coronary syndrome, diabetes, and renal and hepatic dysfunction at the time of surgery; and they had their CRP and albumin levels assessed within 10 days before surgery. Patients with pathological nonmuscle invasive disease at RC were required to have received prior transurethral resection of the bladder tumor (TURBT). The study has been approved by local Ethics Committees and it conforms to the provisions of the Declaration of Helsinki in 1995. Written informed consent to take part was given by all participants.

### Retrieved Data

Demographic data of eligible patients were retrieved along with anthropometric, clinical, and histologic characteristics recorded at the time of surgery, and included the Eastern Cooperative Oncology Group (ECOG) performance status, body mass index (BMI), smoking status, pathologic stage and grade, metabolic syndrome, use of subsequent adjuvant chemotherapy and prior endovesical chemotherapy, presence of concomitant carcinoma in situ (CIS), CRP, and albumin levels. Given the retrospective nature of this study, methods employed for stage and grade assessment, as well as for CRP and albumin levels determination were also retrieved.

### Biochemical, Radiological, and Pathological Assessment

All the institutions involved employed the 2009 tumor-node-metastasis (TNM) staging and the 1973 WHO classification for staging and grading assessment, respectively. Follow-up was homogenous among all institutions involved, and was planned for 5 years after surgery. It included physical examination, abdominal ultrasound, whole body CT scan with and without contrast every 3 to 6 months. Other radiological assessments, such as brain CT /MRI, were evaluated on an individual basis.

### Definition of the Modified GPS

The mGPS was computed as previously described.^18^ Briefly, the scores were assigned using the following criteria: mGPS = 2 for patients with both an elevated CRP level (>1.0 mg/dL) and hypoalbuminemia (<3.5 g/dL); mGPS = 1 for patients with only elevated CRP level (>1.0 mg/dL); for patients with neither CRP nor albumin abnormalities it was assigned a score of 0. Patients with normal CRP levels were assigned an mGPS of 0, irrespective of the serum albumin level.

### Statistical Methods

Descriptive analysis was based on median with range in case of quantitative variables and on frequencies and percentages in case of ordinal and categorical factors. Accordingly, differences between mGPS risk groups were based on Kruskall–Wallis test or the Chi-squared test.

To evaluate the effect of mGPS on survival outcomes (recurrence-free [RFS], overall [OS], and cancer-specific [CSS] survival), survival curves were generated by the Kaplan–Meier method and compared by a log-rank test. To compare the prognostic accuracy of mGPS versus CRP alone, time-dependent receiver operating characteristic curve were used.

In order to adjust the analysis for potential confounding effects and for known prognostic factors a Cox regression model was initially planned; however, due to the violation of the proportional hazard assumption, a weighted Cox regression was applied to obtain unbiased average hazard ratio (HR) estimates.^20^ Results of regression model were reported as HRs with the corresponding 95% confidence intervals and *P* values were based on the Wald test. All statistical tests were 2-sided and *P* value <0.05 were considered to indicate statistical significance.

R statistical software, version 3.2.0 (www.r-project.org), was used for all statistical analyses.

## RESULTS

### Study Population

A total of 1037 patients who had received RC plus pelvic lymphadenectomy were included in the study. Median age at the time of surgery was 70 (range 42–88) years. A total of 676 patients (65.2%) had muscle-invasive disease, while 162 patients (15.6%) had concomitant in situ carcinoma. Positive lymph nodes were found in 266 patients (25.7%), while adjuvant chemotherapy was administered in 301 patients (29.1%). Metabolic syndrome was diagnosed in 273 patients (26.3%), whereas median CRP and albumin levels were 7 mg/L (range 1–23) and 4.4 g/L (range 2.6–5.8), respectively. An mGPS = 0 was observed in 646 patients (62.3%), mGPS = 1 in 297 patients (28.6 %), and mGPS = 2 in 94 patients (9.1%).

### Association Between mGPS and Clinical Characteristics

Clinical characteristics of patients, stratified according to mGPS, are showed in Table 1. Higher mGPS was associated with smoking habit (*P* = 0.030), higher tumor grade (*P* = 0.001), presence of metabolic syndrome (*P* < 0.001), lymph node-positive bladder cancer (*P* < 0.001), use of adjuvant chemotherapy (*P* < 0.001), absence of endovesical chemotherapy (*P* = 0.005), and concomitant CIS (*P* < 0.001). Distribution of age, gender, BMI, and pathological stage did not differ among mGPS categories.

**TABLE 1:**
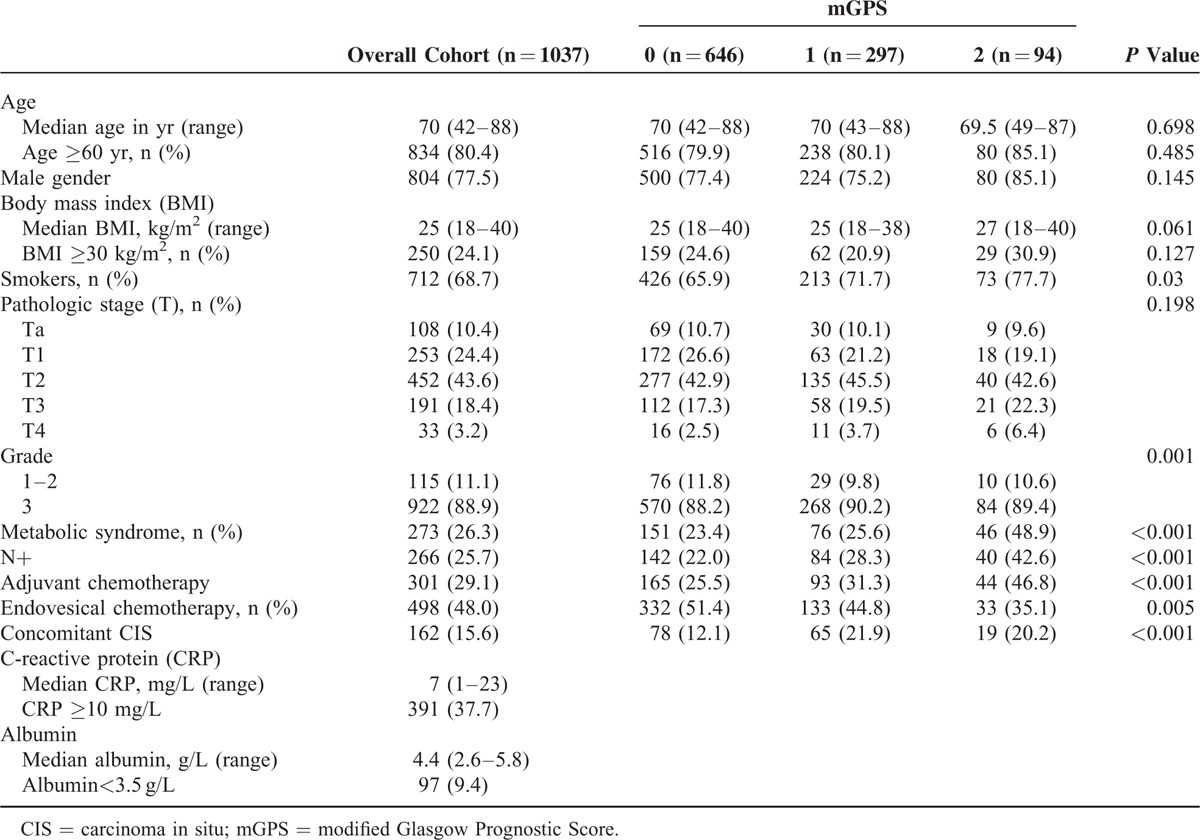
Clinical Characteristics of the Study Population

### Prognostic Role of mGPS on RFS, OS, and CSS

During a median follow-up of 22 months (range 3–60 months), a total of 780 subjects (71.8%) experienced a recurrence of disease. The median RFS time was 23 months (range 21–25 months).

**FIGURE 1:**
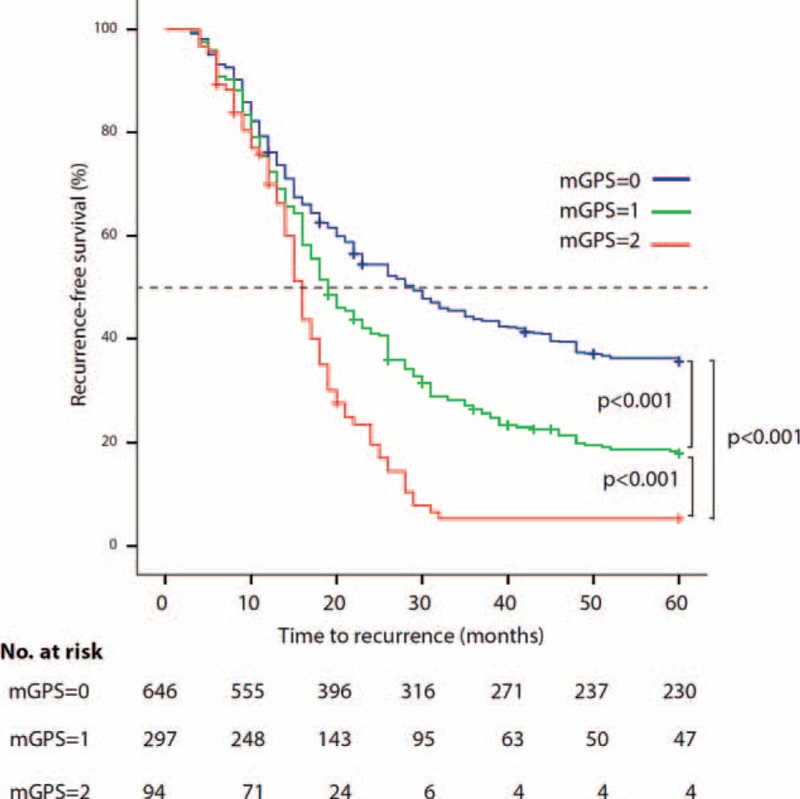
Kaplan–Meier Curve: Preoperative mGPS and RFS in patients with UBC. mGPS = modified Glasgow Prognostic Score; RFS = recurrence-free survival; UBC = urothelial bladder cancer.

In univariate analyses, the mGPS was significantly associated with RFS. In particular subjects with an mGPS equal to 2 had a significantly shorter median RFS compared with subjects with mGPS equal to 1 (16 vs 19 months, HR 1.54, 95% CI 1.31–1.81; *P* < 0.001) or with subjects with mGPS equal to 0 (16 vs 29 months, HR 2.38, 95% CI 1.86–3.05; *P* < 0.001). The log-rank Chi-squared statistics for trend was equal to 64.6 (*P* < 0.001; Fig. 1). The 5-year RFS rates for patients with an mGPS of 0, 1, and 2 were 36% (95% CI 32–40), 18% (95% CI 14–23), and 5% (95% CI 2–14), respectively. When comparing the prognostic accuracy of mGPS versus CRP alone using time-dependent ROC curve analysis, it emerged that mGPS was superior to CRP for predicting a recurrence of disease during the whole follow-up period but the first year (Table 2).

**TABLE 2:**
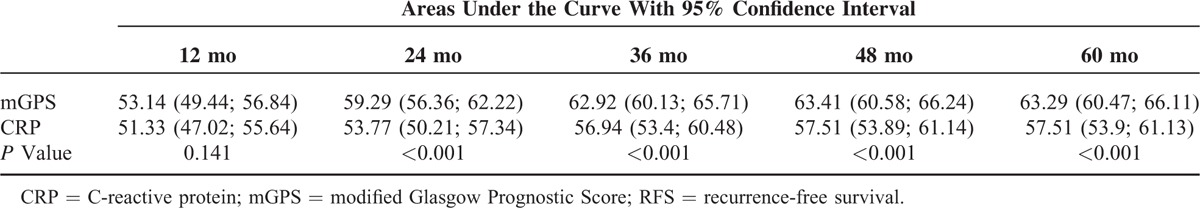
Areas Under the Curve Associated With RFS Using mGPS vs CRP Alone at 12, 24, 36, 48, and 60 Months

The association between mGPS and RFS was confirmed in weighted multivariable Cox model (Table 3). In particular subjects with mGPS equal to 1, had an hazard of recurrence in the first 5 years, 1.2 times higher than subjects with mGPS equal to 0 (95% CI 1.10–1.43; *P* = 0.024). The hazard became even higher in case of mGPS equal to 2 (HR 1.55, 95% CI 1.22–1.98; *P* < 0.001). The other variables that remained predictive of a poorer RFS were increasing age, higher BMI, smoking habit, pathological stage greater than T1, grade 2 or 3, and lymph node-positive bladder cancer.

**TABLE 3:**
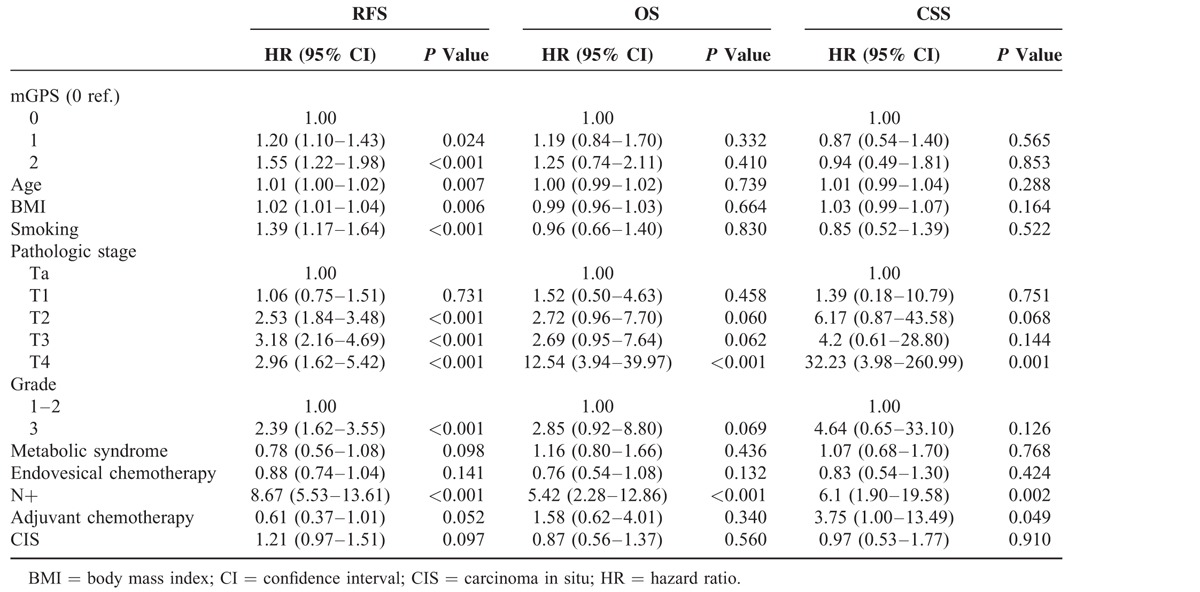
Multivariable Cox Model Showing the Association Between Modified Glasgow Prognostic Score (mGPS) and Recurrence-Free Survival (RFS), Overall Survival (OS), and Cancer-Specific Survival (CSS)

**FIGURE 2:**
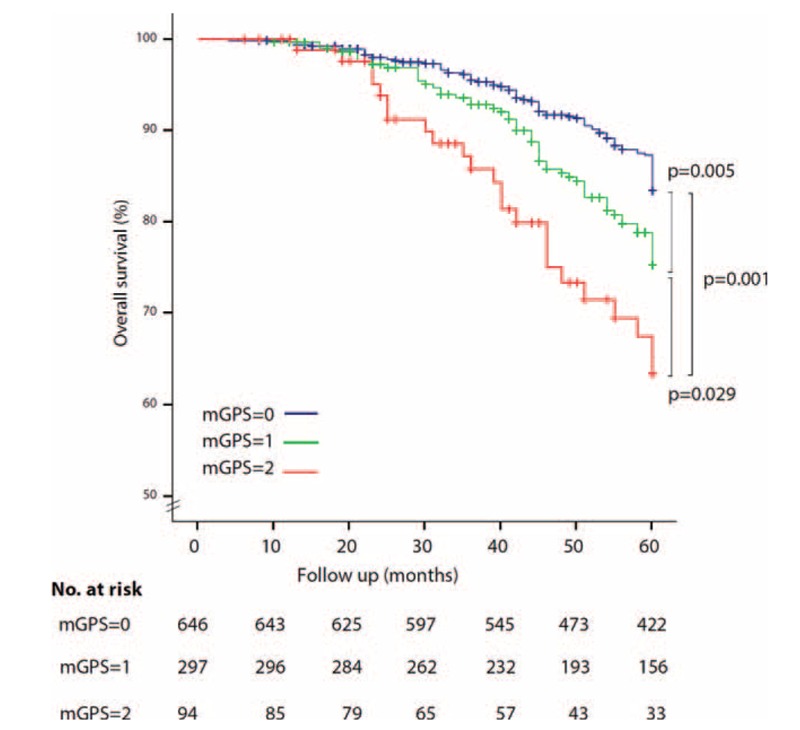
Kaplan–Meier Curve: Preoperative mGPS and OS in patients with UBC. mGPS = modified Glasgow Prognostic Score; OS = overall survival; UBC = urothelial bladder cancer.

**FIGURE 3:**
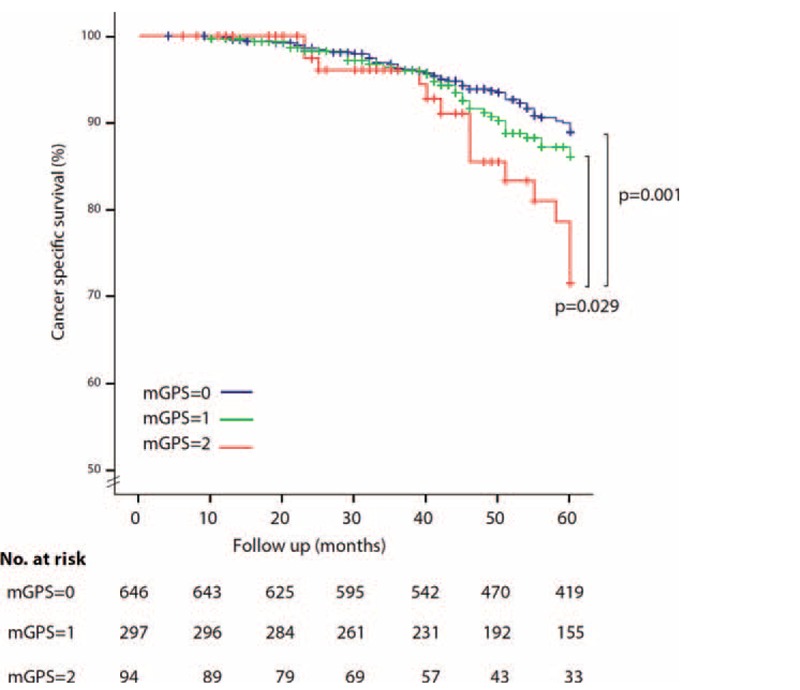
Kaplan–Meier Curve: Preoperative mGPS and CSS in patients with UBC. mGPS = modified Glasgow Prognostic Score; CSS = cancer-specific survival; UBC = urothelial bladder cancer.

Although in univariate analysis higher mGPS was associated with lower OS and CSS (Figs. 2 and 3), this finding was not confirmed at multivariate analysis, which showed that only positive lymph node and T4 stage were predictors of worse prognosis for OS and CSS (Table 3).

## DISCUSSION

The effectiveness of intravesical Bacillus Calmette–Guerin (BCG) shows that local inflammatory responses are essential for control/eradication of UBC,^21^ which is consistent with the association of immunosuppression with bladder cancer.^22^ Conversely, systemic inflammation is detrimental in bladder cancer patients. Nonsteroidal anti-inflammatory medications reduce the risk of UBC, similarly to dietary flavonoids, which have anti-inflammatory and anti-oxidant properties.^23–25^ Among the markers of systemic inflammation, CRP has been extensively investigated in UBC patients undergoing RC. In a retrospective study by Sejima et al^13^ involving 249 consecutive UBC patients treated with RC without neoadjuvant therapy, a positive surgical margin, high CRP (>0.5 mg/dL), low hemoglobin (<10.5 mg/dL), and extravesical T stage (≥pT3a) were independent predictors of poor disease-specific survival. These variables were combined in a risk-group classification model, with patients classified at low, intermediate, and high risk showing a 2/4-year disease-specific survival of 88.0/77.7%, 42.1/23.6%, and 22.1/7.4%, respectively. In the retrospective study by Gakis et al^11^ involving 246 patients undergoing RC for UBC, the 3-year CSS was significantly higher in patients with normal versus elevated CRP (74% vs 44.0%). Elevated CRP, tumor stage, resection margin status, and lymph node density (*P* = 0.02) were independent predictors of CSS. Of note, CRP increased the model predictive accuracy by 4.9% with a concordance index of the final model of 0.788 (*P* = 0.01). A larger retrospective study was conducted by Viers et al,^7^ who analyzed the prognostic role of preoperative NLR in 899 patients undergoing RC, and found that the NLR was associated with greater risks of disease recurrence (HR 1.04; *P* = 0.02), bladder cancer-specific mortality (HR 1.04; *P* = 0.01), and all-cause mortality (HR 1.03; *P* = 0.01). Interestingly, the NLR was also associated with a more advanced pathologic stage (approximately 68% vs 56% of patients with ≤T2 disease had an NLR <2.7 vs >2.7 and approximately 32% vs 44% of patients with >T2 disease had an NLR <2.7 vs >2.7). This result is consistent with those obtained in the series by Sejima et al^13^ and Gakis et al,^11^ which concordantly indicate that levels of CRP are associated with a more advanced disease stage. In our cohort of 1037 patients, we aimed to measure systemic inflammation by using the mGPS, rather than CRP alone. The incorporation of CRP and albumin levels allow to take into account the effects of systemic inflammation and the progressive nutritional decline associated with advanced cancer. We found that a modified mGPS of 1 or 2 (vs 0) was, respectively, associated with a 29% or 79% increase in the HR of recurrence, whereas we did not identify any association of the mGPS with T stage. Unlike the other mentioned studies, we failed to identify any association of the mGPS with OS or CSS at multivariate analysis. This may simply be related to the short follow-up time in a population with no evidence of disease after surgery, with many censored patients at the time of the survival analysis. Interestingly, we found that the mGPS was associated with in situ carcinoma. We found that incidence of in situ carcinoma was approximately double in patients with an mGPS of 1 to 2 versus 0 (21% vs 12%). The diagnosis of in situ carcinoma is elusive and it relies on clinical suspicion (which has low accuracy), urine cytology (which has low sensitivity), and photodynamic diagnosis (which is not universally available).^2^ Pending confirmation in adequately designed studies, the mGPS may help identify those patients who are at increased risk of in situ carcinoma and may benefit from diagnostic random bladder biopsies.

Our study has a number of limitations. Its retrospective and multicenter nature did not allow to adjust the results obtained for the different surgeons’ experience and preferences, as well as for different follow-up schedule and adjuvant chemotherapy indications. Furthermore, a central pathology review was not performed, nor we did not collect data regarding other established markers of systemic inflammation, such as hemoglobin levels and the NLR. The relative prognostic value of these biomarkers was not assessed in the multivariate analysis. Nevertheless, it must be noted that this is the largest cohort of bladder cancer patients ever assessed for a systemic inflammation biomarker, and includes a large proportion of patients with Ta–T1 disease, so the results achieved can be generalized to patients with any stage of the disease. Our study adds evidence to the growing body of literature on the prognostic role of the systemic markers of inflammation. We suggest that mGPS should be explored in prospective clinical trials in order to improve timing of cystectomy in T1 patients, indication of adjuvant chemotherapy in T2 patients, indication for random biopsies to detect CIS.
